# Altered acetyl-CoA metabolism presents a new potential immunotherapy target in the obese lung microenvironment

**DOI:** 10.1186/s40170-022-00292-x

**Published:** 2022-10-26

**Authors:** Spencer R. Rosario, Randall J. Smith, Santosh K. Patnaik, Song Liu, Joseph Barbi, Sai Yendamuri

**Affiliations:** 1 Department of Biostatistics and Bioinformatics, Roswell Park Comprehensive Cancer Center, Buffalo, NY USA; 2 Department of Pharmacology and Therapeutics, Roswell Park Comprehensive Cancer Center, Buffalo, NY USA; 3 Department of Immunology, Roswell Park Comprehensive Cancer Center, Buffalo, NY USA; 4 Department of Thoracic Surgery, Roswell Park Comprehensive Cancer Center, Buffalo, NY USA

**Keywords:** Lung cancer, Metabolism, Obesity, Acetyl Co-A

## Abstract

Contrary to the “obesity paradox,” which arises from retrospective studies relying on body mass index to define obesity, epidemiologic evidence suggests central or visceral obesity is associated with a higher risk for the development of lung cancer. About 60% of individuals at high risk for developing lung cancer or those already with early-stage disease are either overweight or obese. Findings from resected patient tumors and mouse lung tumor models show obesity dampens immune activity in the tumor microenvironment (TME) encouraging disease progression. In line with this, we have observed a marked, obesity-specific enhancement in the presence and phenotype of immunosuppressive regulatory T (Treg) cells in murine tumors as well as the airways of both humans and mice. Leveraging direct metabolomic measurements and robust inferred analyses from RNA-sequencing data, we here demonstrate for the first time that visceral adiposity alters the lung microenvironment via dysregulated acetyl-CoA metabolism in a direction that facilitates immune suppression and lung carcinogenesis.

**Supplementary Information:**

The online version contains supplementary material available at 10.1186/s40170-022-00292-x.

## Introduction

Cancer attributed to obesity has emerged as a major threat to public health, with the incidence rates of a significant fraction of cancers [1] having increased concurrently with increasing obesity incidence [2] and mortality [3]. Consequently, population studies have explored this phenomenon in lung cancer, but contrary to expectations, these studies have associated obesity, as defined by a high body mass index (BMI), with improved patient outcomes [4, 5], giving rise to the “obesity paradox” [6]. However, the use of BMI as a measure of obesity is coming under scrutiny, as it has been shown to introduce misclassification problems that can lead to inappropriate biases that impede our understanding of obesity-related effects [7]. Therefore, in order to resolve our understanding of the obesity paradox, and define interventions aimed at targeting the pro-cancer effects of obesity in lung cancer, we utilize imaging-based measurements of visceral fat, a more accurate measure of obesity [8]. Our recent studies indicate obesity is associated with worse survival outcomes in lung cancer if obesity is defined by abdominal visceral fatness. Further, increased visceral adiposity has been associated with a worse prognosis in lung cancer patients undergoing chemotherapy [9], indicating an underlying biological etiology associated with visceral fat. Visceral, centrally deposited, fat is defined by its enhanced metabolic activity, as compared to peripheral subcutaneous fat [10, 11]. Also, visceral adipose tissue secretes several adipokines and cytokines leading to a proinflammatory, procoagulant, and insulin-resistant state collectively known as the metabolic syndrome [8, 12]. Visceral fat is more strongly associated with an adverse metabolic risk profile, even after accounting for the contribution of other standard anthropometric indices [13]. These systemic effects exerted by visceral adiposity are presumedly involved in cancer biology [14] and are a major research area given known impacts on all cancer hallmarks [15], including dysregulated tumor metabolism and the inflammatory microenvironment. While much is known about obesity in the context of other solid tumors, the impact of obesity on lung cancer is underexplored [16], with proposed mechanisms, including alterations of tumor metabolism and leptin-mediated immune modulation [17].

To date, much of the research surrounding obesity-induced metabolic changes in cancer is focused on enriched lipidomic profiles. Beyond lipid and fatty acid abundance and desaturation, the elongation of fatty acid chains has been identified as a prominent feature of lung tumors [18]. However, in other malignancies like breast cancer, obesity has been linked with altered glucose metabolism [19] and enhanced mitochondrial oxidative phosphorylation [20]. Some studies have also shown that the intracellular metabolism of immune cells is also deregulated by the lipid-rich environment in obesity [21] with potential implications for their anti-tumor activity [22]. While it is known that obesity impacts systemic metabolism to alter the tumor microenvironment (TME), how this may influence lung cancer progression, metastasis, and anti-tumor immunity [22], and specific immune cell populations, is not fully understood.

One such immune population of interest is regulatory T (Tregs) cells. While there is limited evidence from patient samples implicating Tregs in lung cancer development, several mouse model studies show enrichment of Tregs in pre-cancerous lung tissues and link this accumulation to eventual tumor burden. For example, exposure to tobacco carcinogens in a study using the NNK/AJ mouse model of chemically induced lung cancer demonstrated increased Tregs in the lung within a week of exposure (and prior to tumor development) [23]. Interestingly, in this same study, Kras transgenic mice lacking Tregs developed significantly fewer lung tumors than Treg-competent mice. In another lung cancer study using the same model, depletion of lung-associated Tregs with an activated, effector-like phenotype reduced tumor burden [24], illustrating (1) the pro-tumor potential of activated Tregs and (2) the value of targeting these cells to alter the outcome. Indeed, immunosuppressive CD4^+^Foxp3^+^ regulatory T (Treg) cells with a surface marker profile high in activation markers and proliferative indicators (e.g., high KI67 expression) tend to be the dominant Treg subset recovered from tumors and increases in the relative fraction of these activated or effector-like tumor Tregs is associated with poor outcomes in many cancers [25, 26]. To this end, it has also been previously shown that Treg pools, which play a key role in regulating inflammatory and metabolic responses in visceral adipose tissue (VAT), are heavily impacted by obesity. In fact, dysregulated inflammation in the adipose tissue, marked by increased proinflammatory T cell accumulation and reduced Tregs, contributes to obesity-associated insulin resistance [27]. However, the molecular mechanisms underlying T cell-mediated inflammation in the adipose tissue remain largely unknown [28]. Predicted mechanisms include skewing adaptive immunity in the visceral adipose tissue, thereby contributing to diet-induced obesity (DIO) and insulin resistance, and tissue inflammation modulation via adaptive and innate immune mechanisms [29]. Previous experiments have indicated that obese adipose Treg depletion stems from reduced local differentiation rather than impaired homing, resulting in adipose inflammation [30]. The biological etiology underlying this phenomenon has not been thoroughly studied. Additionally, few studies have simultaneously explored the metabolic impacts of obesity on the tumor, tumor microenvironment, and immune cell populations in lung carcinogenesis.

While recent studies have sought to uncover metabolic changes that occur with lung cancer progression [31], an understanding of how obesity may also impact the metabolome in these patients is lacking. The link between obesity and cancer can be attributed in part to the state of chronic inflammation that develops in obesity. For example, acetyl-CoA production and protein acetylation patterns are highly sensitive to metabolic state and are significantly altered in obesity [32]. In the present study, we observed a strong association between acetyl-CoA metabolism and obesity-related effects in not only lung tumors, but also the immune microenvironment. Multiple changes in gene expression and metabolites involved in acetyl-CoA-related pathways were observed in the settings of obesity, exacerbated lung cancer progression in patients and mice. Transcriptional analysis of Tregs recovered from obese mouse tumors also revealed several probable changes linked to acetyl-CoA metabolism. Patient airway gene expression patterns also linked these same pathways to the development of lung cancer and paired RNA-sequencing and metabolomics analysis of obese mouse lungs drew clear associations between altered acetyl-CoA-relevant metabolism and an immunological niche permissive to tumorigenesis. Specifically, obesity-associated enhancements in Treg abundance, activation, and markers of both functional potency and phenotypic stability in the murine airway were associated with alterations in the biosynthetic commitment of acetyl-CoA to general and Treg-specific processes (i.e., acetylation of polyamines for export and the post-translational modification FoxP3). These findings suggest a novel association between a specific element of the obesity dysregulated metabolic landscape and immune suppression during the development and progression of lung cancers.

## Results

### Transcriptional dysregulation of metabolic pathways associated with acetyl-CoA metabolism are altered in obesity in human and mouse lung cancers

**Fig. 1:**
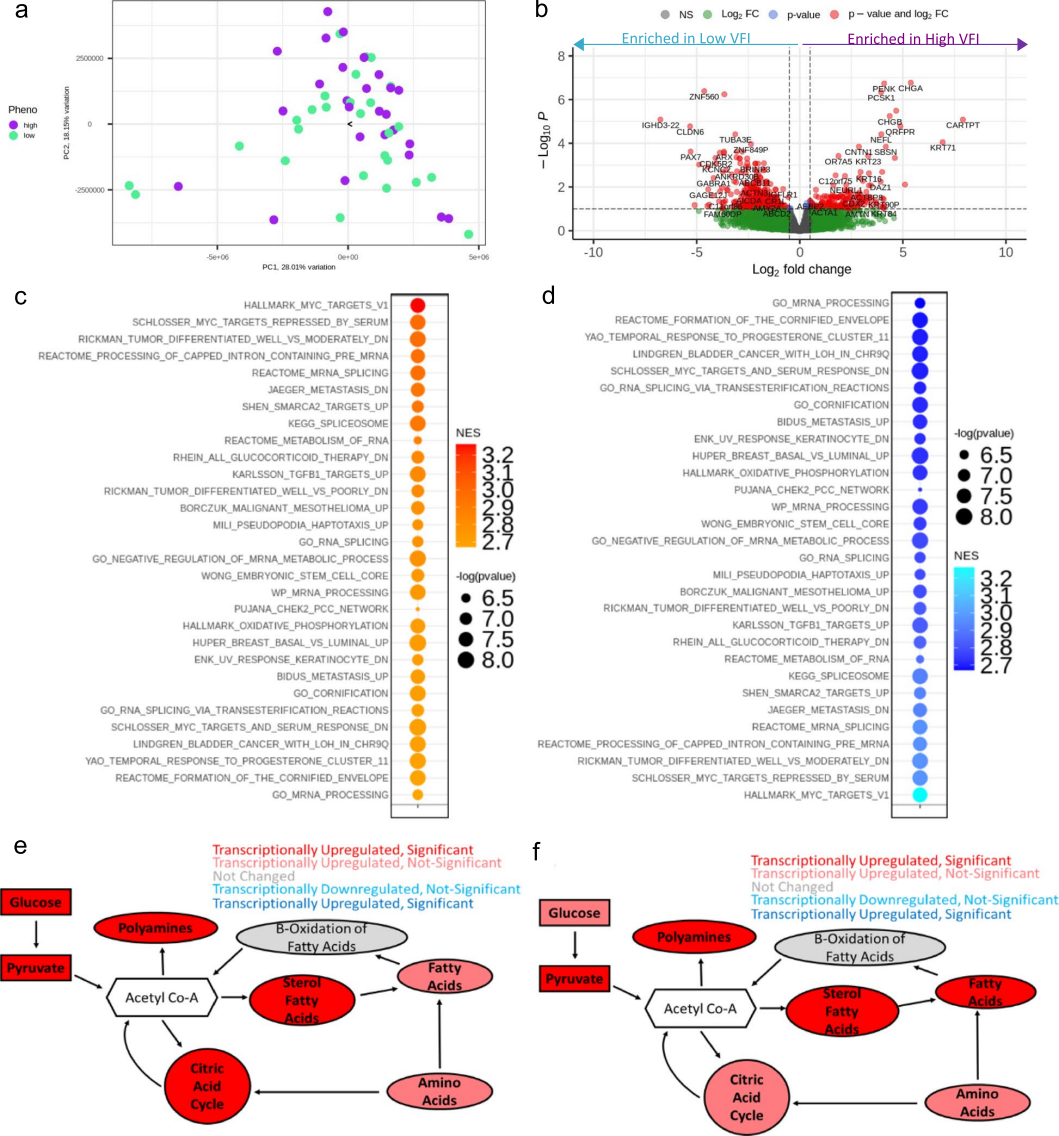
Obesity impacts transcriptional Acetyl Co-A metabolism in humans, and this is recapitulated in mice similarly. (**a**) ORIEN patient data, when split into high visceral fat (top tertile) as compared to low visceral fat (lowest tertile) separate in a PCA and (**b**) reveal several significantly down-regulated (red, left) genes and upregulated (red, right) genes. GSEA of upregulated (**c**) and downregulated (**d**) genes reveal several pathways of interest such as cell cycle, epigenetics, immune, and metabolism. Pathways that result in the increased production and consumption of Acetyl Co-A, are transcriptionally upregulated (red) to a statistically significant extent (darker red, *p* < 0.05) in obese as compared to non-obese tumor tissues in both human ORIEN data (**e**) and these findings were confirmed in the transcriptional data from the tumors of LLC mice (**f**)

Obesity is associated with metabolic disturbances, primarily in lipid and carbohydrate metabolism [33], both of which have been shown to impact tumor development [34], and immune cell function [35]. To elucidate metabolic dysregulation associated with obesity in human lung cancer, we utilized RNA-sequencing data from NSCLC patients with quantified visceral adiposity (visceral fat index; VFI) using the Oncology Research Information Exchange Network (ORIEN). We compared the top tertile (high VFI) to the lowest tertile (low VFI) and saw mild patient sample separation via principal component analysis (Fig. 1a). Comparing these two groups, we identified 274 differentially expressed genes, 186 of which were downregulated/enriched in low VFI and 88 of which were upregulated/enriched in high VFI (adjusted *p* value < 0.05, |logFC|>1.5) (Fig. 1b). Gene set enrichment analysis of differentially expressed genes (Fig. 1c, d) and resulted in enrichment of genes associated with aggression (e.g., metastasis, differentiation, and stemness), immune (interleukin 12 production and lymphocytes), metabolism (e.g., RNA metabolism, oxidative phosphorylation, and progesterone response), and oncogenes (e.g., MYC) in both high and low visceral fat. Given our focus on understanding metabolic reprogramming that underlies obesity, we then applied our novel bioinformatics pipeline [36], to determine which of 114 metabolic pathways were most highly significantly transcriptionally dysregulated in patients with high VFI (top tertile) vs. those with lower VFI (bottom tertile). This revealed obesity-related transcriptional metabolic dysregulation in expected pathways, given the literature (carbohydrate and fatty acid metabolism), and unexpected pathways (folate one-carbon metabolism and the polyamine biosynthetic pathway), which all converge on acetyl-CoA flux (Fig. 1e). Further, applying the same bioinformatics pipeline, which allows for cross-species comparison, we confirmed these findings of transcriptional metabolic dysregulation in subcutaneous (s.c.) Lewis lung carcinoma (LLC) tumors harvested from either normal-weight mice or those with diet-induced obesity generated by feeding with normal chow and a high-fat diet, respectively prior to tumor cell implantation. As in obese patients, in this controlled system, tumors from obese mice were similarly transcriptionally upregulating carbohydrate, lipid, and one carbon/polyamine biosynthesis (Fig. 1f). Upon modeling the metabolic pathway dysregulation observed in these tumors, it was revealed that a majority of the metabolic pathway dysregulation occurring in the obese tumor microenvironment was centered around acetyl-CoA metabolism. This, therefore, highlights a need to further interrogate the importance of this pathway in the context of lung carcinogenesis.

### Significant metabolic dysregulation associated with acetyl-CoA metabolism is observed in obesity-related tumor growth

**Fig. 2:**
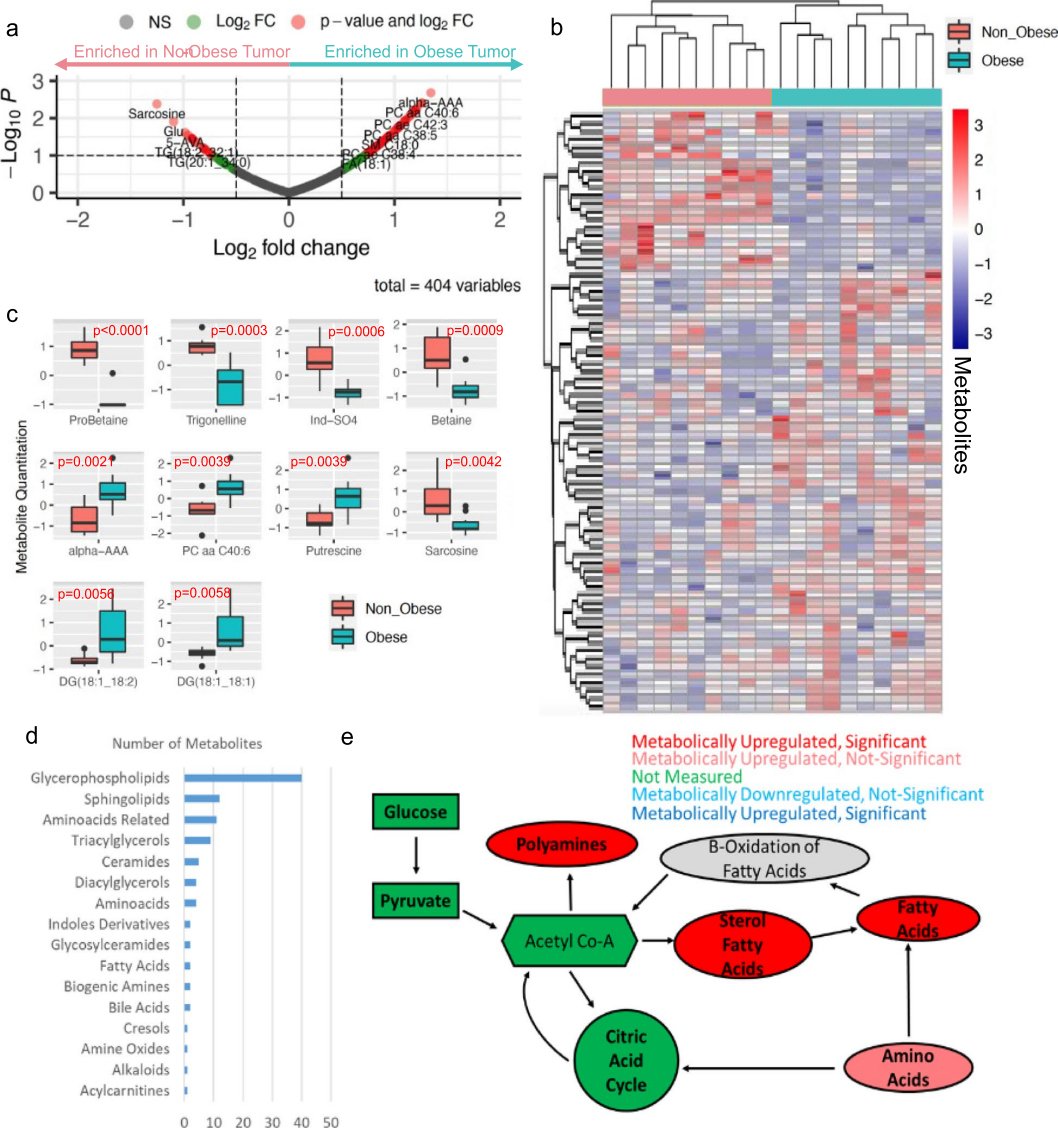
Obesity impacts acetyl Co-A metabolism in mice. (**a**) Biocrates metabolomics assay revealed 100 metabolites that were altered in the tumors of obese LLC mice, as compared to non-obese mice (**b**) which separates normal diet (pink) from diet induced obesity mice (teal) lungs, based on the expression of these metabolites. (**c**) Boxplots of the top 10 differential metabolites shows significant differences spanning several biochemical classes (*p* from Limma output). (**d**) Quantification of metabolites by biochemical classes reveals glycerophospholipids are the most highly dysregulated metabolite classes (**e**) Mapping of these metabolites, revealed upregulation of pathways consuming Acetyl Co-A (red) to a statistically significant extent (dark red, *p* < 0.05)

To look at the metabolic dysregulation that occurs at the metabolite level, LLC tumors, from obese and normal-weight mice (*n*=10/group) were subjected to metabolomics analysis on the Biocrates MXP Quant500 panel [37]. This analysis revealed over 100 differential metabolites (*p*<0.1) between obese and normal-weight tumors (Fig. 2a, Supplementary Table 1), which separated samples by adiposity based on Euclidean distance, in the resulting heatmap shown in Fig. 2b, which emphasizes distinct patterns of metabolite expression between normal and obese lung tumor growth. Further, examination of the top 10 differential metabolites shows significant separation between the normal and obese tissues (Fig. 2c), with many being lipids. Overall, these 100 differential metabolites span 16 different biochemical classes (Fig. 2d), the largest of which being the Sphingolipids and Glycerophospholipids, which utilize acetyl-CoA for their production. Further, amino acids and biogenic amines were highly significant differential between the two groups. While acetyl-CoA was not directly measured in this assay, we were able to track acetyl-CoA flux, which has been shown to affect tumor development [38], metabolism in the tumor microenvironment [39], and compromised T cell function, and more specifically TCR signaling [40]. Here, the results of the metabolomics studies implicate several pathways (Fig. 2e) metabolically altered in association with obesity, which overlap with the transcriptional findings in both human and mouse tissues.

### Obesity enhances the frequencies, activation, and characteristic gene expression of the murine lung-associated Tregs

**Fig. 3:**
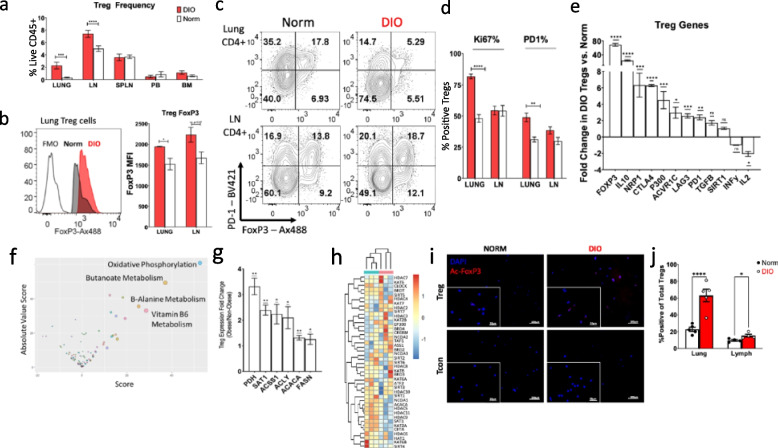
Acetyl Co-A related transcriptional metabolic dysregulation in obese T-regs. Six-week old C57BL/6 mice were fed a high fat diet or normal chow for 14–16 weeks to generate obese (DIO) mice and normal (norm) controls, respectively (*n*= 5–6 mice/group). Single-cell suspensions of tissue-infiltrating leukocytes were generated, and the frequencies of Tregs among the recovered leukocytes (CD45+ cells) were found by flow cytometry (**a**) representative flow plots of lung and lymph nodes, (**b**) the levels of Foxp3 markers of activated, (**c**) “effector” Tregs PD-1 and (**d**) Ki67 were found by flow cytometry. (**e**) Expression of Treg-associated mRNAs by lung Tregs was also analyzed by qRT-PCR and fold change in DIO vs. norm controls are shown. (**f**) Pathways that result in increased metabolic activity (OxPhos) and acetyl Co-A metabolism (butanoate metabolism) are dysregulated in Tregs from obese vs. non-obese LLC (node size = -log(pVal), increased score/absolute value score = more transcriptionally dysregulated). (**g**) Expression of select metabolic genes by Tregs isolated from the lungs of obese and non-obese mice. Foxp3+/CD25+/CD4+ Tregs were recovered from the lung tissues of C57BL/6 mice fed either a high fat (obese) or control diet (non-obese) by FACS for mRNA isolation. Levels of the indicated transcripts were measured by RT-PCR. Shown are mean fold changes in gene expression in obese vs. non-obese Tregs. (**h**) Acetyl-CoA transcriptional signature shows separation between obese and non-obese Tregs. Sorted Treg and Tcon cells were slide-immobilized by Cytospin and subjected to proximity ligation assay to observe levels of acetylated FOXP3 protein (red) and nuclei counter stained with DAPI (blue). (**i**) Representative 20x images, with insets at 40X and (**j**) the mean proportion of Tregs with acetylated Foxp3 across 10–20X fields (~200 cells) are shown. Shown are mean values ±SEM or representative flow plots (panels **b** and **c**) from 2–7 independent experiments. In panel **j** Error bars depict the SEM. *N*=5 mice/group. Ac-Foxp3 = acetylated Foxp3

In prior studies, we found that diet-induced obesity was associated with suppressed immune activity in the murine tumor microenvironment coincident with elevated frequencies of intra-tumoral Tregs expressing high levels of activation markers (CD44, ICOS) and suppressive mediators (PD-1, Lag3) [41]. Interestingly, obesity was also linked to a Treg pool in the mouse airway that is enhanced both in terms of its relative size, and expression of markers indicating an activated and highly suppressive phenotype. Assessing the effects of obesity on the immune contexture of various murine tissues by flow cytometry revealed that diet-induced obesity (DIO) significantly elevated the abundance of FoxP3+ Tregs with leukocyte pools, particularly in lung and lymph node cells (Fig 3a). Compared to normal weight (norm) controls, these DIO Tregs displayed increased levels of FOXP3 (Fig. 3b), and PD-1 and KI67 (Fig. 3c, d, respectively), markers known to be upregulated on activated, effector-like “eTregs” that accumulate in the tumor niche [42, 43]. FACS-based purification of Tregs from control and obese cohorts of FoxP3-GFP reporter mice followed by gene expression analysis by RT-PCR revealed that a number of transcripts characteristically upregulated in, or important for Tregs and eTregs (*Foxp3*, *Il10*, *Nrp1*, *Ctla4*, etc.; Fig. 3e). These apparent enhancements in suppressor cell populations and phenotype likely contribute to the prevalent immune dysfunction reported by us and others in obese mice and patients. Yet, the mechanisms responsible remain to be uncovered. To shed light on the potential involvement of the pathways scrutinized above in this phenomenon, we set out to better understand the transcriptional metabolic differences that occur in Treg pools as a result of obesity.

### Acetyl-CoA metabolism-associated pathways altered in tumor and lung-infiltrating Tregs in obese mice

Given the known impact that the metabolism of the tumor and tumor microenvironment can have on T cell function [39], paired with evidence that suggests obese lungs undergo immune dysregulation characterized by indications of immune inactivity, we first wanted to show these effects were particularly exacerbated in Tregs before assessing altered metabolism. To this end, we challenged obese and non-obese cohorts of Foxp3-gfp reporter mice with s.c. LLC tumor cells as in prior studies [41]. GFP+ Tregs were recovered from the leukocytes infiltrating the obese and non-obese tumors (*n*=3/group) by FACS for sequencing. We then specifically assessed the metabolic immune milieu in Tregs of obese tumors. While Tregs represent an important immune cell population, little is known about the metabolic dependencies of this population [44]. Consequently, it is necessary to study the specific metabolic requirements of Tregs and how obesity impacts this underlying metabolic requirement. Once again, applying the transcriptional metabolic pipeline, we revealed significant transcriptional dysregulation of several novel carbohydrate- and lipid-related pathways, such as cyclooxygenase arachidonic acid metabolism and butanoate metabolism (Fig. 3f), all of which impact acetyl-CoA pools. To this end, it was important to also understand how acetyl-CoA metabolism is impacted in non-tumor-bearing lung tissue, prior to cancer development, for intervention purposes. Previous studies have shown human lung tumors possess higher levels of polyamines than surrounding tissues [45] and increased levels of polyamines in the plasma/urine are biomarkers of poorer prognosis [46]. More recent investigations have revealed, SAT1, which utilizes acetyl-CoA to acetylate polyamines, was enhanced in pathogenic Th17, and suppressed in Treg cells. Further, chemical and genetic perturbation of polyamine metabolism results in the inhibition of Th17 cytokines and promotes Foxp3 expression [47]. To determine whether obesity affects key players in acetyl-CoA metabolism of lung Tregs, obese, and non-obese Foxp3-GFP reporter mice were utilized. Lung tissues were excised and digested prior to isolation of Tregs by FACS and gene expression analysis by RTPCR. In obese non-tumor bearing tissue Tregs, in addition to the upregulation of acetyl-CoA metabolic transcripts, we found increased expression of many acetyl-CoA-related transcripts, including SAT1, as compared to non-obese non-tumor bearing tissue Tregs (Fig. 3g). Further, to elucidate potential impacts on histone acetylation, we applied a 43-transcript histone acetylation-related gene signature, comprised of a 40-gene signature [48] with the addition of SAT1, ACACA, and ASS1. This signature resulted in strong separation via clustering of obese and normal Tregs, which indicates that obesity is potentially driving a distinct acetyl-signature (Fig. 3h), which further provides evidence that obesity may impact acetyl-CoA pools via altered acetyl-CoA metabolism.

It is known that FOXP3 protein is subject to acetylation, a post-translational modification that stabilizes both the transcription factor’s expression and activity [49–51] requiring acetyl-CoA [51]. Suspecting that the dysregulated acetyl-CoA expression patterns in obese lung infiltrating Tregs may allow for enhanced levels of FOXP3 acetylation, a proximity ligation assay (PLA) to assess acetylated FOXP3 protein in Tregs [52] was performed on Tregs from the lungs of DIO and Norm Foxp3 reporter mice. Indeed, a significantly higher proportion of Tregs display acetylated FOXP3 proteins in the DIO lung and to a lesser extent the lymphoid tissues of these mice (Fig. 3i, j). This finding is very much in line with our observations of enhanced Foxp3+ Treg presence in the DIO lung (Fig. 3a–c), and it supports the notion that obesity alters cellular acetyl-coA pools and processes dependent on this molecule. Corroborating our PLA findings, we also observed significant upregulation of transcripts encoding mediators of FOXP3 acetylation (i.e., p300, SIRT1) in DIO Treg relative to norm controls (Fig. 3e). In all, these results strongly suggest that altered acetyl-coA metabolism may promote Treg-mediated immune suppression in the immune milieu of the obese.

### Progression of airway precancerous lesions is associated with acetyl-CoA metabolic alterations

**Fig. 4:**
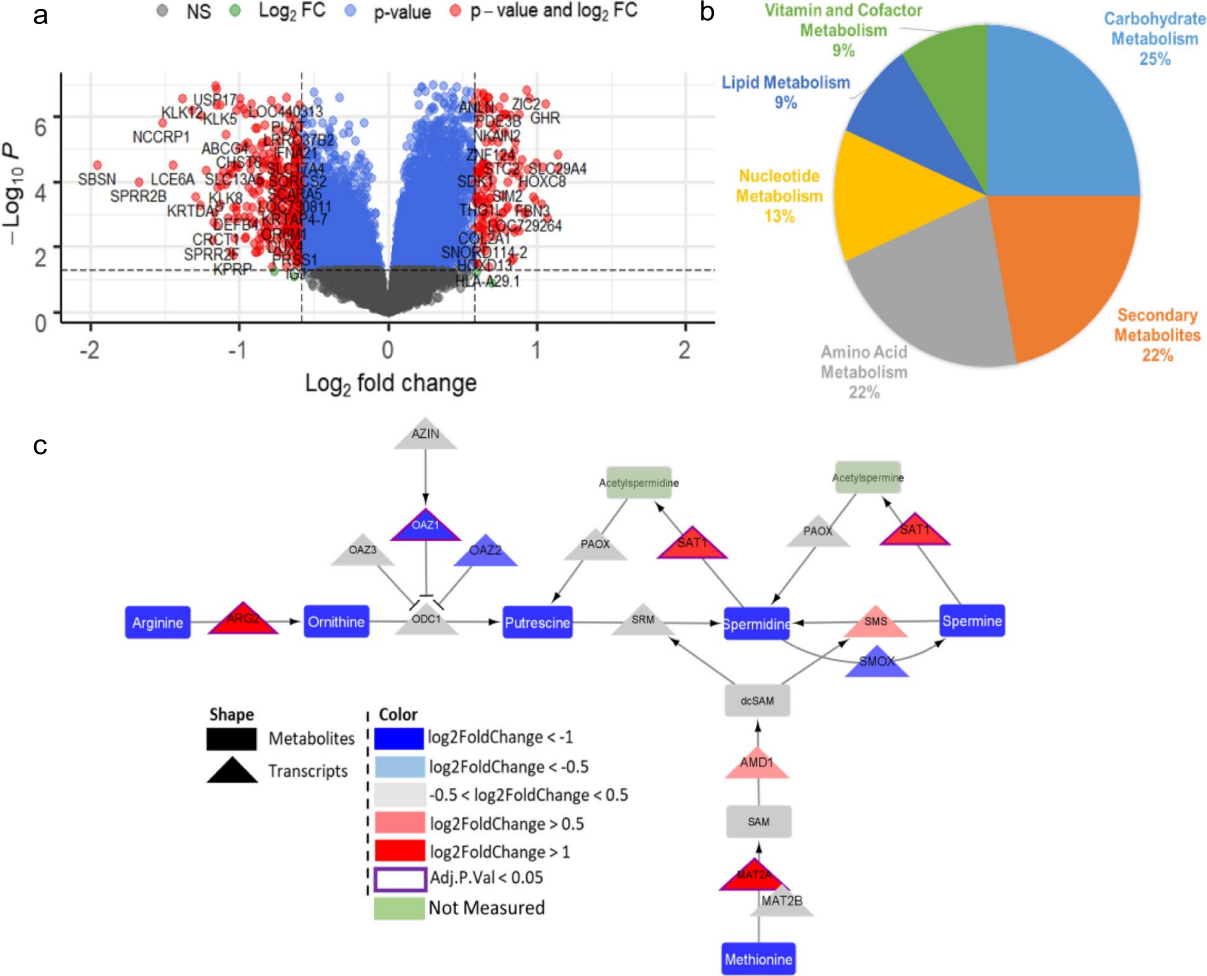
Acetyl Co-A related dysregulation in precancerous human and mouse lungs. (**a**) RNA-sequencing of biopsied lesions that either progressed or regressed, highlighted over 400 metabolites that were significant by both adjusted p value and fold change (red), which are relatively evenly distributed between up- (right) and down- (left) regulated genes. (**b**) Metabolic pipeline analysis of these DEGs resulted in large amounts of transcriptional dysregulation in carbohydrate (25% of significantly dysregulated pathways), amino acid (22% of significantly dysregulated pathways), and lipid/lipid-related pathways (21% of significantly dysregulated pathways – Lipid Metabolism and the lipid related pathways in Secondary Metabolites). (**c**) Complementary integration of paired RNA-sequencing and metabolomics in non-tumor bearing lungs of obese and non-obese mice demonstrated increased transcription (red triangles) but decreased metabolite levels (blue rectangles) of the polyamine pathway in the obese lung, suggesting utilization of Acetyl Co-A pools for acetylation of polyamines (not measured here) for exportation

We utilized the post-cancer development data, to better understand which metabolic changes are most relevant within the pre-cancerous milieu, and therefore may represent putative biomarkers of interest for future studies. Here, we utilized microarray data from lung carcinoma in situ (CIS) pre-cancerous lesions [53]. In this study, half of these lesions progressed to invasive cancer (*n*=17), and half regressed, never progressing to cancer (*n*=16). We performed differential gene expression analysis with the goal of deriving a molecular signature of progression to lung cancer. This analysis revealed 462 DEGs (adjusted *p* value < 0.05, |logFC|>1.5) between progressive and regressive precancerous lesions (Fig. 4 a). Further, we performed the same metabolic pipeline analysis [36] and enriched several classes of metabolic pathways that were transcriptionally dysregulated between the progressive and regressive patient samples, including pathways associated with lipid, carbohydrate, and amino acid metabolism (Fig. 4b). Many of these metabolic pathways were previously enriched in human tumor tissue, with acetyl-CoA being a central metabolite to them all. To confirm the utility of mouse models in understanding the precancerous state, we utilized paired RNA-sequencing and metabolomics (Biocrates) in the non-tumor-bearing lungs of obese and non-obese mice (*n*= 5/group). Like the human RNA-sequencing data, we found several dysregulated pathways dependent on acetyl-CoA utilization, for the production of downstream metabolites. One such pathway was the polyamine biosynthetic pathway (Fig. 4c). Here, we see increased transcription (red triangles) with decreased basal polyamines (blue rectangles), in obese compared to non-obese lungs. This, with increased transcriptional levels of SAT1, point to enhanced utilization of acetyl-CoA for the acetylation of polyamines.

Together, these findings suggest a role for obesity-dysregulated acetyl-CoA metabolism in the facilitation of carcinogenesis operative in the tumor and pre-cancerous lesions, as well as the immune milieu in the obese lung. This also represents a novel point of metabolic intervention that may be leveraged to prevent progression to cancer or allow for more effective immune-mediated control of established tumors. Further, unlike other cancer types, acetyl-CoA metabolism has not yet been thoroughly studied in obese, pre-cancerous lungs which represent different metabolic opportunities for cancer prevention.

## Discussion

Altered metabolism is a hallmark of cancers, dating back to the Warburg Effect, first observed in 1930 [54], and includes other pathways like shifted oxidative phosphorylation and glutaminolysis, as a means of increasing biomass production and diverting carbon sources into biosynthetic pathways that promote sustained tumor proliferation [55]. Therefore, metabolic inhibitors represent promising anti-cancer drugs, and their use has dated back to the 1940s for treating acute leukemia in children [56]. These drugs have been shown to result in high levels of toxicity, partially given that many other cell types heavily rely on the same metabolic pathways. For example, metabolic profiles of cancer cells and activated T lymphocytes are similar, raising the risk of metabolic inhibitors impairing the immune system [57]. While this can typically be detrimental to immune function in controlling tumor growth, in the obese setting, immune cells are functioning aberrantly and therefore also need to be targeted. Recently, Ringel et al. showed that with obesity, tumor cells increase uptake of fat, whereas tumor-infiltrating CD8+ T cells do not, and further that blocking metabolic reprogramming of tumor cells improves anti-tumor immunity, suggesting that interventions that exploit metabolism to improve cancer immunotherapy [22]. It is worth noting that while the authors of this study did assess markers of activation and functionality on Tregs in obese tumors, they did not find a prominent effect of obesity on the levels of Tregs. It is possible that variables associated with the animal model highlighted, specialty diet formulation, and the duration of high-fat diet administration may result in a shift in the prevailing cell types and metabolic pathways chiefly responsible for obesity-associated mechanism of immune suppression. This further suggests a need to better understand and identify targets that can be co-targeted in the tumor and T cells, with obesity. While looking at CD8+ T cells is important in the context of disease, it is also important to understand other immune cell populations.

Overall, the data presented in this manuscript reveal that metabolism is highly dysregulated with obesity and may facilitate lung carcinogenesis and altered Treg metabolism. Given similarities between altered metabolism between tumors and pre-cancerous lesions in the obese setting, targeting these metabolic pathways, which seemingly hinge on acetyl-CoA metabolism, which has impacts on both metabolism as a whole, and the epigenome, which may provide new, preventative treatment options, especially in obese, former smokers. Despite the emerging studies that have indicated both supporting and antagonizing roles for obesity in lung cancer, we have demonstrated this depends heavily on how obesity is defined in this setting (BMI or VFI [7, 8, 12, 13]). Interestingly, we saw consistent metabolic alterations in several lipids and carbohydrate metabolic pathways in obese pre-cancerous, tumor, and Treg pools. Hence, our data argue that different populations of cells within a heterogeneous obese tissue may all benefit from a single therapeutic point of leverage aimed at altered acetyl-CoA metabolism and thereby acetyl pools. Indeed, targeting pathways in the obese setting that either produce acetyl-CoA (glycolysis, citric acid cycle, butanoate metabolism) or consume acetyl-CoA (polyamine catabolism, one carbon folate metabolism, fatty acid biosynthesis) may help to halt the progression of pre-cancerous lesions to cancerous lesions in the obese patient, as long as obesity is measured by VFI. These findings start to define the unique metabolic alterations that occur as a result of obesity in lung cancer and begin to remedy the “obesity paradox” in this disease setting. However, it may be important to consider the absolute levels of acetyl-CoA in these tissues, which we have not done yet.

We hypothesize that acetyl-CoA pools, in particular, are important for eliciting pro-tumorigenic effects in the context of obesity; however, the effects of depleting this pool altogether may hinder cellular bioenergetic capacity in healthy tissues as well. Therefore, identifying key production and consumption pathways, like the polyamine biosynthetic pathway, will be key to optimize preventative therapeutic options for these patient populations. We also recognize that these studies are not without their limitations. First, we are solely focused on metabolic alterations occurring specifically in the Treg populations. Future studies should focus on other immune cell populations, which may also play a role in carcinogenesis and therapeutic response, like macrophages [58], which are impacted by visceral adiposity [59]. Therefore, in the future, we plan to not only assess the transcriptional and metabolic alterations that occur in the tumor, normal tissue, and Tregs, but also within the entirety of the immune milieu. Further, we would like to confirm many of our results through the use of extensive human samples, at varying stages of the disease, to explore consistency in metabolic alterations that occur with obesity both prior to and throughout lung cancer development. However, we believe this research provides evidence that acetyl-CoA metabolism should be seriously studied more thoroughly for therapeutic intervention in the obese lung carcinoma setting.

## Materials and methods

### Publicly available datasets

For the query of lesions that either progressed or did not progress, data can be found at GSE109743. Differential expression analysis was conducted using DESeq2 [60]. Further, differentially expressed genes underwent transcriptional metabolic assessment [36], to determine which metabolic pathways are most highly transcriptionally dysregulated to a statistically significant extent.

### Mice and diet-induced obesity

Obese wild-type C57BL/6 male mice fed a high-fat diet (HFD) and age- and sex-matched normal diet controls were purchased from the Jackson Laboratory. Foxp3-DTR-GFP reporter mouse founders (C57BL/6 background; first generated in the lab of Dr. Alexander Rudensky [61]) were also obtained from the Jackson Laboratory and bred in-house. Six-week-old male offspring were fed a high-fat diet (60 kcal% fat, 5.2 kcal/gram; Bio-Serv product # S3282). In all experiments, HFD was administered for at least 14 weeks to induce obesity. Normal weight controls were age- and sex-matched mice fed a conventional chow diet (10 kcal% fat, 3.8 kcal/gram, Bio-Serv product #S4031) in parallel. All mice were housed in a specific pathogen-free facility, and all procedures were approved by the Institutional Animal Care and Use Committee.

### Subcutaneous tumor implantation and monitoring

The Lewis lung carcinoma (LLC) cell line was purchased from ATCC and passaged in vitro. For tumor challenge experiments, 1×10^5^ tumor cells were injected subcutaneously (s.c.) into the flanks of obese and normal-weight mice. After monitoring tumor growth (volume) for ~16–21 days, post-implantation mice were euthanized, and tumor tissues were harvested. Tumor sections were frozen for subsequent RNASeq and Biocrates analysis, and in some experiments, tumor-infiltrating Tregs were recovered from Foxp3-GFP reporter mice by FACS (Aria II, BD) for RNASeq analysis.

### Analysis of Tregs by flow cytometry

Lung tissues were collected and digested using a collagenase/hyaluronidase cocktail (Stem Cell Technologies) and mechanical dissociation (GentleMACs, Miltenyi Biotec). Single-cell suspensions of these and cells harvested from lymph nodes of the indicated mice were made in a staining buffer (1×10^6^ cells/20μl) containing fluorescently labeled antibodies recognizing relevant surface markers (CD45 Alexa-700, clone I3/2.3; CD4 BV785/PerCP-Cy5.5, clone L3T4; CD8 BUV395, clone 53-6.7; CD25 BV605/Alexa-647, clone PC61; PD-1 BV421, clone 29F.1A12) after labeling of non-viable cells with LD-Blue (molecular probes). Intracellular staining of FOXP3 (Alexa-488, clone 150D/E4) and Ki67 (Alexa-647, clone 11F6) was performed with a FOXP3/transcription factor fixation/permeabilization kit (eBiosciences) as per the manufacturer’s protocol. Data (1–3×10^6^ events/sample) were collected on an LSR2 cytometer using FACS-DIVA software (BD Biosciences). Results were analyzed using Flowjo (V10.8) software, and population frequencies and mean fluorescent intensity (MFI) of phenotypic markers were found.

### Lung tissue Treg isolation and analysis

Obese and non-obese cohorts of Foxp3-GFP reporter mice were generated as described above. Lung tissues were collected and digested using a collagenase/hyaluronidase cocktail (Stem Cell Technologies) and mechanical dissociation (GentleMACs, Miltenyi Biotec). Single-cell suspensions from DIO and Norm C57/BL6 FoxP3-GFP reporter mice lungs and lymphoid tissue (peripheral lymph nodes and spleen) were enriched for CD4+ cells with a magnetic bead isolation kit (Dynabeads Untouched Mouse CD4 Isolation Kit; Thermo-fisher) and stained with a viability dye and fluorescently labeled antibodies recognizing Treg-defining surface markers prior to high-speed sorting (FACS) using an either an Aria II (BD) or a SONY MA-900. Viable Tregs (FOXP3-GFP+) and conventional CD4+ “Tcon” cells (GFP-) were then quantified and used for downstream applications.

### Proximity ligation assay

Viable Tregs and Tcon cells isolated as described above were subjected to proximity ligation assay as described [62] to measure the acetylation of FoxP3. Briefly, sorted cells (1–5×10^4^ cells) were centrifuged onto glass slides (Cytospin), fixed with periodate-lysine-paraformaldehyde, permeabilized with 0.2% Triton X-100, and stained with anti-mouse FoxP3 (Clone:MF14, BioLegend) and anti-rabbit acetylated lysine (Clone:RM101, Abcam). Slides were subsequently treated with DuoLink in situ PLA probes kit (Sigma) as per the manufacturer’s instructions. Nuclei were counterstained with DAPI. Cells were imaged using a ZOE fluorescent microscope (BioRad), and an Axioscope 5 (Zeiss) and the proportions of DAPI+ cells positive for the FoxP3-specific acetylated lysine signal was found via ImageJ.

### Quantitative real-time PCR

Tregs recovered from lung tissues were directly lysed and converted to cDNA using the “Cells to CT” kit (Thermo) following the manufacturer’s protocol. Generated cDNA was subjected to quantitative real-time PCR using Power-Up SYBR green master mix (Applied Biosystems) in a CFX96 thermocycler (Bio-Rad). Primers for indicated genes were previously verified and selected from the PrimerBank database (ref). Results from DIO-derived cells were normalized to housekeeping gene RPL13a and further compared to gene expression levels in norm mice; displayed as log(2) fold change. All primers used in these analyses were obtained from IDT, and sequences are listed in Supplementary Table 2.

### RNA sequencing

The total RNA from the tissue was isolated by TRIzol reagent. Samples were analyzed in the Roswell Park Comprehensive Cancer Center Genomics Shared Resource. TruSeq Stranded mRNA (Illumina, San Diego, CA, USA) was used for library preparation. Raw sequencing reads were aligned to the human genome (GRCh38.74) and mouse genome (Mm10) using STAR [63] (40.4×10^6^ –69.0×10^6^ uniquely mapped reads per sample). Raw feature counts were normalized, and differential expression analysis was conducted using DESeq2 [60]. Differential expression rank order was utilized for subsequent Gene Set Enrichment Analysis (GSEA), performed using the clusterProfiler package in R. Gene sets queried included the Hallmark, canonical pathways, and GO Biological Processes Ontology collections available through the Molecular Signatures Database (MSigDB) [64]. Further, differentially expressed genes underwent transcriptional metabolic assessment [36], to determine which metabolic pathways are most highly transcriptionally dysregulated to a statistically significant extent.

### Metabolomics—Biocrates assays

Tumor tissue of age- and sex-matched obese (*n*=10) and non-obese (*n*=10) mice were obtained at ~16–21 days post-implantation. Samples were prepared and analyzed in the Roswell Park Comprehensive Cancer Center Bioanalytics, Metabolomics and Pharmacokinetics Shared Resource, using the MxP Quant 500 kit (Biocrates Life Sciences AG, Innsbruck, Austria) in accordance with the user manual. Tumor samples were homogenized in a ratio of 1 mg of tissue to 3 μL of solvent (85% ethanol and 15% 0.01 M phosphate buffer) using the optimized setting on the Omni-Bead Ruptor 24 (Omni International, Kennesaw, GA). The homogenate was centrifuged to obtain a supernatant which was added to the plate. Ten microliters of each supernatant, quality control (QC) sample, blank, zero sample, or calibration standard were added to the filter spot (already containing internal standard) in the appropriate wells of the 96-well plate. The plate was then dried under a gentle stream of nitrogen. The samples were derivatized with phenyl isothiocyanate (PITC) for the amino acids and biogenic amines and dried again. Sample extract elution was performed with a 5-mM ammonium acetate in methanol. Sample extracts were diluted with either water for the HPLC-MS/MS analysis (1:1) or kit running solvent (Biocrates Life Sciences AG) for flow injection analysis (FIA)-MS/MS (50:1), using a Sciex 5500 mass spectrometer. Data was processed using MetIDQ software (Biocrates Life Sciences AG), and Limma [65] for differential metabolite analysis, and to obtain *p* values for this analysis.

### Modeling with cytoscape

Pathway maps were generated using the Cytoscape software [66], specifically the VizMapper functions. Pathway maps were adapted from existing pathway maps in WikiPathways [67]. DESeq2 output for DEG analysis was utilized to direct shading of genes within the pathway: red (positive fold change, statistically significant), blue (negative fold change, statistically significant), or gray (non-statistically significant), for individual cancer site transcripts (triangles) and metabolites (rounded rectangles).

## Supplementary Information

**Additional file 1: Table S1 and S2**. Cancer metabolism.

## Data Availability

All data generated and/or analyzed in this current study are either available on the Gene Expression Omnibus (GSE provided) or are available from the corresponding author upon reasonable request.
